# *Trypanosoma brucei* TbIF1 inhibits the essential F_1_-ATPase in the infectious form of the parasite

**DOI:** 10.1371/journal.pntd.0005552

**Published:** 2017-04-17

**Authors:** Brian Panicucci, Ondřej Gahura, Alena Zíková

**Affiliations:** 1 Biology Centre, Czech Academy of Sciences, Institute of Parasitology, Ceske Budejovice, Czech Republic; 2 Faculty of Science, University of South Bohemia, Ceske Budejovice, Czech Republic; Hunter College, CUNY, UNITED STATES

## Abstract

The mitochondrial (mt) F_o_F_1_-ATP synthase of the digenetic parasite, *Trypanosoma brucei*, generates ATP during the insect procyclic form (PF), but becomes a perpetual consumer of ATP in the mammalian bloodstream form (BF), which lacks a canonical respiratory chain. This unconventional dependence on F_o_F_1_-ATPase is required to maintain the essential mt membrane potential (Δψm). Normally, ATP hydrolysis by this rotary molecular motor is restricted to when eukaryotic cells experience sporadic hypoxic conditions, during which this compulsory function quickly depletes the cellular ATP pool. To protect against this cellular treason, the highly conserved inhibitory factor 1 (IF1) binds the enzyme in a manner that solely inhibits the hydrolytic activity. Intriguingly, we were able to identify the IF1 homolog in *T*. *brucei* (TbIF1), but determined that its expression in the mitochondrion is tightly regulated throughout the life cycle as it is only detected in PF cells. TbIF1 appears to primarily function as an emergency brake in PF cells, where it prevented the restoration of the Δψm by F_o_F_1_-ATPase when respiration was chemically inhibited. *In vitro*, TbIF1 overexpression specifically inhibits the hydrolytic activity but not the synthetic capability of the F_o_F_1_-ATP synthase in PF mitochondria. Furthermore, low μM amounts of recombinant TbIF1 achieve the same inhibition of total mt ATPase activity as the F_o_F_1_-ATPase specific inhibitors, azide and oligomycin. Therefore, even minimal ectopic expression of TbIF1 in BF cells proved lethal as the indispensable Δψm collapsed due to inhibited F_o_F_1_-ATPase. In summary, we provide evidence that *T*. *brucei* harbors a natural and potent unidirectional inhibitor of the vital F_o_F_1_-ATPase activity that can be exploited for future structure-based drug design.

## Author summary

Enzymes are catalysts that drive both a forward and reverse chemical reaction depending on the thermodynamic properties. F_o_F_1_-ATP synthase is a multiprotein enzyme that under normal physiological conditions generates ATP. However, when respiration is impeded, this rotary molecular machine reverses and hydrolyzes ATP to pump protons and maintain the essential mitochondrial membrane potential. While this activity is exceptional in most eukaryotic cells, the unique composition of the *Trypanosoma brucei* mitochondrion dictates that the infectious stage of this human parasite is utterly dependent on the hydrolytic activity of F_o_F_1_-ATPase. While searching for better chemotherapeutics against Human African Trypanosomiasis, several trypanocidal compounds were determined to interact with this enzyme, but they indiscriminately inhibit both the ATP hydrolytic and synthetic activities. A more promising approach involves the conserved eukaryotic protein IF1, a unidirectional inhibitor that prevents just ATP hydrolysis. Auspiciously, we identified this protein homolog in *T*. *brucei* (TbIF1) and its expression is tightly regulated between life stages of the parasite. Importantly, the introduction of exogenous TbIF1 protein specifically inhibits F_o_F_1_-ATPase and is lethal for the infectious stage of *T*. *brucei*. Therefore, we have identified a natural inhibitor of an essential and druggable enzyme that can be exploited for future structure-based drug design.

## Introduction

*Trypanosoma brucei rhodesiense* and *T*. *b*. *gambiense* are the flagellated protists responsible for Human African Trypanosomiasis in 36 sub-Saharan African countries where the tsetse fly insect vector resides. While vector control projects have greatly reduced the number of new cases (<3,000 annually) since the last epidemic subsided in the late 1990’s, an alarming number of these extracellular parasites are demonstrating resistance to current drugs that already possess efficacy and toxicity problems, in addition to their complex treatment regimens. Furthermore, salivarian *Trypanosoma* species (e.g. *T*. *b*. *brucei*, *T*. *vivax*, *T*. *congolense*) cause disease in livestock, namely nagana in cattle, which inflict substantial economic burdens in rural areas where agricultural development is a necessity. For these reasons, there is a renewed urgency to develop a new generation of therapeutics based upon the unique *T*. *brucei* biological processes that have already been characterized [1].

A striking feature of *T*. *brucei* is the ability to rapidly adapt its metabolism while alternating between the glucose-rich blood of a mammalian host and the abundant proline found in the hemolymph and tissue fluids of the blood-feeding tsetse fly [2,3]. These different carbon sources dictate a shift in bioenergetics that manifests in the unique architecture and activities of the singular mitochondrion. The *T*. *brucei* insect stage or procyclic form (PF) utilizes a branched mitochondrion that is fully developed: composed of many cristae, eight Krebs cycle enzymes used for both anabolic and catabolic reactions in a partitioned cycle and respiratory chain complexes I, II, III and IV [4]. The enzymatic complexes III and IV pump protons into the mitochondrial (mt) intermembrane space, generating a membrane potential (Δψm) that is coupled to ATP production by the F_o_F_1_-ATP synthase [5,6]. This rotary molecular machine converts the potential energy of the proton gradient into the chemical energy of ATP when protons are allowed to flow down their energy gradient by passing through the proton pore located in the membrane-embedded F_o_ domain. This action drives the rotation of the asymmetrical central stalk within the matrix protruding F_1_ catalytic domain, which undergoes three sequential conformational states that result in the synthesis of ATP.

In contrast, the streamlined mitochondrion of the mammalian bloodstream form (BF) lacks a functional cytochrome *c* mediated respiratory chain and the parasite must rely on glycolysis to provide sufficient energy for all of the cellular processes occurring during this life stage [7]. Although BF cells have a reduced mitochondrion, it still harbors vital processes like Fe-S cluster assembly, mt fatty acid synthesis, acetate production, Ca^2+^ homeostasis and RNA editing [8–11]. These functions are performed by proteins that are encoded in the nuclear genome and imported into the mitochondrion, a process that absolutely requires a Δψm [12]. Since respiratory complexes III and IV are absent in BF trypanosomes, the Δψm is maintained by the reverse activity of F_o_F_1_-ATP synthase, which hydrolyzes ATP and translocates protons into the mt intermembrane space [13]. This ATPase activity is also essential for dyskinetoplastic (Dk) trypanosomes (i.e. *T*. *b*. *evansi*, *T*. *b*. *equiperdum*), which lack all or critical portions of their mt DNA [14]. Without a full complement of mt DNA, these parasites do not express the F_o_F_1_-ATPase subunit a, a critical component of the proton pore. Thus, dyskinetoplastic strains have acquired one of several different compensatory mutations that enables the catalytic F_1_-ATPase to energize the inner mt membrane by coupling ATP hydrolysis with the electrogenic exchange of ADP^3-^ for ATP^4-^ by the ATP/ADP carrier (AAC) [15,16].

While this ATP hydrolase activity must be unrelenting in these infectious trypanosomes, it is only observed under specific conditions in other eukaryotes (i.e. during oxygen deprivation or in response to damaged mt respiratory chain complexes). On the rare occasion that mt respiration becomes compromised and the Δψm collapses, the rotation of F_o_F_1_-ATP synthase reverses and acts as an ATP-consuming proton pump. However, this unrestrained hydrolysis of ATP replenishes the Δψm so inefficiently, that it actually accelerates cell death due to the depletion of ATP [17]. Thus, specific F_o_F_1_-ATPase small molecule inhibitors are currently being developed to prevent human tissue damage during ischemia [18].

Intriguingly, eukaryotic cells already possess a specific inhibitor of the F_1_-ATPase activity in the form of a nuclear encoded small protein, inhibitory factor 1 (IF1) [19]. The IF1 protein is highly conserved throughout evolution, with homologs found in plants, yeast, worms and vertebrates [19–22]. Since its discovery, many details have been gleaned about the molecular mechanism responsible for the interaction of IF1 with F_1_-ATPase [23,24]. *In vitro*, this protein effectively inhibits the intact F_o_F_1_-ATPase from hydrolyzing ATP, but in the presence of a proton gradient, IF1 is not able to impede ATP synthesis [25]. Hence, unlike most other inhibitors, IF1 is a unidirectional inhibitor that only blocks the ATP hydrolytic activity of the F_o_F_1_-ATP synthase. *In vivo*, the activity of IF1 is dependent upon the acidification of the mt matrix, which occurs when mt respiration is compromised. Thus, it has been assumed that the physiological role of IF1 is to act as an emergency brake that prevents the futile hydrolysis of ATP by the F_o_F_1_-ATPase [26,27]. However, recent studies have suggested that IF1 can also play a role in the metabolic reprogramming observed during cellular differentiation and in some types of cancer [28,29].

Due to the ability of IF1 to inhibit the hydrolytic activity of F_o_F_1-_ATPase and its potential to selectively impact the viability of the infectious stage of *T*. *brucei*, we identified the IF1 homolog in this human pathogen and characterized its effect on the cellular growth and mt physiology in both parasitic life stages.

## Methods

### Plasmid preparation

The TbIF1 (Tb927.10.2970) RNAi construct, which targets all 345 bp of the mRNA coding sequence, was generated with a PCR product amplified from *T*. *brucei* strain 427 genomic DNA with the following oligonucleotides: Fw—CAC AAG CTT ATG CTG CCC CTC CGT GT, Rev—CAC CTC GAG TTG CTT CTC GTT CGT TAA C. Utilizing HindIII and XhoI restriction sites inherent in the primers (underlined), this fragment was cloned into the pZJM plasmid [30], which contains head-to-head, tetracycline (tet)-regulated T7 promoters. For the tet inducible expression of an ectopic TbIF1 fused with a C-terminal 3x V5 tag, the TbIF1 open reading frame was PCR amplified (Fw: ACA AAG CTT ATG C GC CGT GTA TC, Rev: CAC GGA TCC TTG CTT CTC GTT CGT TAA C) and cloned into the pT7_V5 vector using HindIII and BamHI restriction enzymes [31].

### Trypanosoma culture conditions and generation of cell lines

The wildtype PF *T*. *brucei* Lister 427 strain and tet-inducible PF 29–13 cells were grown *in vitro* at 27°C in SDM-79 medium containing hemin (7.5 mg/ml) and 10% fetal bovine serum (FBS) [32]. Meanwhile, the following cultures were maintained at 37°C with 5% CO_2_ in HMI-9 media containing 10% FBS: wildtype BF *T*. *brucei* Lister 427, the derived BF single marker (SM) strain [32], laboratory induced dyskinetoplastic *T*. *bruce*i Dk164 [33] and genetically modified Dk *T*. *b*. *evansi* cells [34]. The PF 29–13, BF SM and *T*. *b*. *evansi* cell lines constitutively express the ectopic bacteriophage T7 RNA polymerase and the prokaryotic tet repressor, which allows for the tet inducible expression of either ectopic V5-tagged proteins or dsRNA. As described previously [32], the appropriate cells were then transfected with NotI linearized pZJM or pT7_V5 plasmids containing the TbIF1 gene. Both plasmids were targeted to the rDNA intergenic spacer region. The addition of 1 μg/ml of tet into the media triggers either the induction of RNAi or the expression of tagged TbIF1. Throughout the analyses, a Z2 Cell Counter (Beckman Coulter Inc.) was used to measure cell densities in order to maintain the cultures within their exponential mid-log growth phase of 1x10^6^ to 1x10^7^ cells/ml for PF and between 1x10^5^ to 1x10^6^ cells/ml for BF cells.

### SDS-PAGE and western blot

Protein samples were separated on SDS-PAGE, blotted onto a PVDF membrane (PALL) and probed with the appropriate monoclonal (mAb) or polyclonal (pAb) antibody. This was followed by incubation with a secondary HRP-conjugated anti-rabbit or anti-mouse antibody (1:2000, BioRad). Proteins were visualized using an ECL system (Biorad) on a ChemiDoc instrument (BioRad). The PageRuler prestained protein standard (Fermentas) was used to determine the size of detected bands. Primary antibodies used in this study were: mAb anti-V5 epitope tag (1:2000, Invitrogen), mAb anti-mtHsp70 (1:2000), pAb anti-APRT (1:1000). Native TbIF1 antigen was purified (see below) and was sent to Davids Biotechnologie (Regensburg, Germany) to produce a polyclonal antiserum (1:1000).

### Digitonin subcellular fractionation

Whole cell lysates (WCL) were prepared from *T*. *brucei* PF 29–13 and PF 29–13 cells with an ectopic V5-tagged TbIF1 that were either induced for 48 hours or never induced. SoTe/digitonin fractionation was performed as follows: 1x10^8^ cells were harvested by centrifugation, washed in PBS-G, resuspended in 500μl SoTE (0.6 M Sorbitol, 2 mM EDTA, 20 mM Tris-HCl pH 7.5) and lysed with 500μl SoTE containing 0.03% digitonin. The cells were then incubated on ice for 5 minutes before centrifugation (4°C, 7000 rpm, 3 min). This allowed us to separate the supernatant, consisting of the cytosolic subcellular fraction (CYTO), from the pellet, which represents the organellar fraction (ORG) of the parasite. These enriched fractions along with WCL were resolved by SDS-PAGE and analyzed by immunoblotting.

### Immunofluorescence assay

*T*. *brucei* subcellular localization of the overexpressed TbIF1 V5-tagged protein was determined by an immunofluorescence assay that amplified the signal of a monoclonal anti-V5 antibody (Life Technologies) with a FITC-conjugated secondary anti-mouse antibody (Sigma). Co-localization was verified using Mitotracker RED (Invitrogen), a dye that stains mitochondria in live cells and is well-retained after fixation. DAPI (4,6-diamidino-2-phenylindole; Sigma) treatment was used to visualize nuclear and mitochondrial DNA. The images of the stained cells and their fluorescence were captured with a Zeiss Axioplan 2 fluorescence microscope.

### *In situ* Δψm measurement

Estimation of the Δψm *in situ* was done spectrofluorometrically using the indicating dye safranine O (Sigma). *T*. *brucei* PF cells (2x10^7^ cells/ml) were resuspended in a reaction buffer containing: 200 mM sucrose, 10 mM HEPES-Na (pH 7.0), 2 mM succinate, 1 mM MgCl_2_, 1 mM EGTA and 1 mM ATP. The reaction was activated with digitonin (50 μM), while NaCN (50 μM), oligomycin (2.5 μg/ml) and FCCP (5 μM) were injected at specific time points throughout the assay. Changes in the amount of fluorescence over time were detected on an Infinite M200 microplate reader (TECAN) (excitation = 496 nm; emission = 586 nm). Values were normalized according the following equation: $\text{normalized }\left(e_{i}\right)=\;\;\frac{e_{i}-E_{min}}{E_{max}-E_{min}}$

E_min_—the minimum value for variable E

E_max_—the maximum value for variable E

### *In vivo* Δψm measurement

The Δψm was determined by utilizing the red-orange fluorescent stain tetramethylrhodamine ethyl ester (TMRE, Life Technologies). PF and BF cells maintained within their exponential growth phase were stained with 60 nM of the dye for 30 min at 27°C or 37°C, respectively. Cells were pelleted (1300 g, 10 min, RT), resuspended in 2 ml of PBS (pH 7.4) and immediately analyzed by flow cytometry (BD FACS Canto II Instrument). For each sample, 10000 events were collected. Treatment with the protonophore FCCP (20 μM) was used as a control for mt membrane depolarization. Data were evaluated using BD FACSDiva (BD Company) software.

### ATPase assays

The ATPase activity was measured by two different methods. The first assay utilizes the Sumner reagent to detect the release of free phosphate when ATP is hydrolysed [35]. Briefly, crude mt lysates were obtained from 2x10^8^ cells by SoTe/digitonin extraction (0.015% digitonin, 0.6 M Sorbitol, 2 mM EDTA, 20 mM Tris-HCl pH 7.5). Mt pellets were resuspended in an assay buffer (200 mM KCl, 2 mM MgCl_2_, Tris-HCl pH 8.0) and the addition of ATP to a final concentration of 5 mM initiated the 20 min reaction. Where indicated, samples were pre-treated with the F_1_-ATPase specific inhibitor sodium azide (2 mM) for 10 min. The 100 μl enzymatic reactions were deproteinated by adding 1.9 μl of 70% perchloric acid. After a 30 min incubation on ice, the samples were spun down (16000g, 10 min, 4°C) and 90 μl of the supernatant was incubated for 10 min with 0.5 ml of the Sumner reagent (8.8% FeSO_4_.7H_2_O, 375 mM H_2_SO_4_, 6.6% (NH_4_)Mo_7_O_24_.4H_2_O). 200 μl was then transferred to a 96 well plate and the absorbance was measured at 610 nm using a microplate reader (Infinite M200Pro, Tecan). To calibrate the assay, a standard curve was calculated from the absorbance values of linear inorganic phosphate samples (0–2 mM).

The ATPase activity was also measured spectrophotometrically by an ATP regenerating assay [36], wherein the hydrolysis of ATP is determined indirectly by coupling this activity to the oxidation of NADH by lactate dehydrogenase, resulting in a quantifiable decrease in the absorbance of NADH at 340 nm. The assay mixture contained 15 μg of purified mitochondria lysed with 1% dodecyl maltoside, 50 mM Tris-HCl (or 50 mM MOPS-NaOH for pH values under 7.0), 50 mM KCl, 2 mM MgSO_4_, 0.2 mM NADH, 2 mM ATP, 1 mM phosphoenolpyruvate (PEP), 5 μl/ml pyruvate kinase from rabbit muscle (PK; Sigma-Aldrich) and 5 μl/ml lactate dehydrogenase from bovine heart (LDH; Sigma-Aldrich). All measurements were performed in 1 ml spectroscopic cuvettes with the reaction mixture incubated at 37°C.

### ATP production assay

ATP production was measured as described previously [37,38]. Briefly, crude mt preparations from PF cells were obtained by digitonin extraction. ATP production was then activated by adding the oxidative phosphorylation substrates ADP (67 μM) and succinate (5 mM). The resulting concentrations of ATP were determined by using the ATP Bioluminescence Assay Kit HS II (Roche) and a microplate luminometer (Orion II). To determine how much of the synthesized ATP was due to oxidative phosphorylation, specific inhibitors against succinate dehydrogenase (6.7 mM malonate) and the ADP/ATP carrier (33 ug/ml atractyloside) were incubated with the enriched mitochondria samples for 10 min on ice prior to the start of the assay.

### Cloning, expression, and purification of recombinant TbIF1

The gene fragment encoding TbIF1 without its predicted mt localization signal and stop codon was PCR amplified from PF *T*. *brucei* strain 427 genomic DNA with forward and reverse primers containing *Nde*I and *Hind*III restriction sites, respectively. The 3’-terminal primer included sequence encoding a hexahistidine tag. The digested PCR products were then ligated into the *Nde*I/*Hind*III linearized expression vector pRUN [39]. The verified plasmid was used to transform the *Escherichia coli* expression strain C41(DE3) [40], which was grown in LB medium. When the OD_600_ reached ~ 0.4, the expression of the recombinant protein (rTbIF1) was induced by the addition of 1 mM IPTG. After three hours, the cells were harvested, washed in PBS and resuspended in ice-cold buffer A (20 mM Tris-HCl pH 7.4, 10% (w/v) glycerol, 100 mM NaCl, 25 mM imidazole) supplemented with the complete EDTA-free protease inhibitor cocktail (Roche). The bacteria were then lysed with lysozyme (75 μg/ml) for 30 min at 4°C in the presence of DNase I (15 U/ml). The lysate was sonicated (5 x 20 s with 1 min on ice between pulses), cleared by centrifugation (15000 g, 30 min, 4°C), filtered through a syringe filter (0.45 μm) and loaded onto a HisTrap nickel affinity column using the AktaPrime chromatography system (GE Healthcare). The column was thoroughly washed with buffer A before the rTbIF1 protein was eluted with a 50 ml linear gradient of buffer A containing imidazole ranging from 20 to 500 mM. SDS-PAGE analysis identified the fractions containing rTbIF1, which were pooled and dialyzed against 20 mM Tris-HCl pH 7.4 and 10% glycerol. Finally, the purified protein was concentrated to 10–20 mg/ml (PES, MWCO 3.5 kDa, Sartorius, Germany), flash-frozen in liquid N_2_ and stored at -80°C.

## Results

### *T*. *brucei* genome contains an IF1 homolog

**Fig 1:**
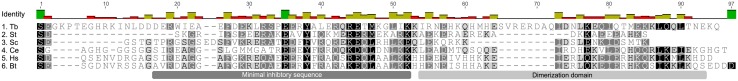
TbIF1 shares homology with IF1 proteins from various model organisms. Using the alignment program MAFFT, the amino acid sequence of IF1 from *T*. *brucei* (Tb) is compared to those of *Solanum tuberosum* (St, [20]), *Saccharomyces cerevisiase* (Sc, [22]), *Caenorhabditis elegans* (Ce, [21]), *Human sapiens* (Hs, [44]) and *Bos taurus* (Bs, [19]) IF1s. Identical and conservatively substituted resides are shaded in black and grey, respectively. Predicted mt import signal sequences were removed from each coding sequence prior to the analysis. The minimal inhibitory sequence [42] and dimerization domain of bovine IF1[45] are indicated. The green, yellow, and red bars above the alignment indicate when the nucleotides at each position have identities of 100%, between 100% and 30%, and <30%, respectively.

IF1 is widespread throughout the eukaryotic kingdom, with homologs found in plants, yeast, worms and vertebrates. To search for a *T*. *brucei* IF1 homolog within the TriTrypDB database (www.tritrypdb.org), we employed a reciprocal blastp search using the yeast inhibitory protein Inh1 and bovine IF1. The putative candidates were further analyzed using HHpred toolkit (http://toolkit.tuebingen.mpg.de), a program based on the comparison of hidden Markov models that utilize structure prediction to identify homologous relationships. These analyses revealed that only the translated product of the candidate gene Tb927.10.2970 (TbIF1) truly resembled yeast Inh1 and bovine IF1. TbIF1 is annotated as a conserved hypothetical protein with a calculated molecular mass of 13.8 kDa, which includes the 23 N-terminal residues predicted by Mitoprot II [41] to comprise the mt targeting sequence. A MAFFT alignment with IF1 proteins from different organisms depicts a conserved region within TbIF1 (residues 17–52) that corresponds to the minimal inhibitory domain of its bovine ortholog [42] (Fig 1). The identity and similarity of this entire bovine minimal inhibitory sequence with the putative TbIF1 is only 26% and 45%, respectively. These metrics are even less pronounced when comparing the C-terminal region, which has been characterized as a oligomerization domain in the bovine IF1 that regulates activity of the inhibitor [43].

### TbIF1 localizes to the PF mitochondrion, but is not expressed in cells dependent on F_1_-ATPase activity

**Fig 2:**
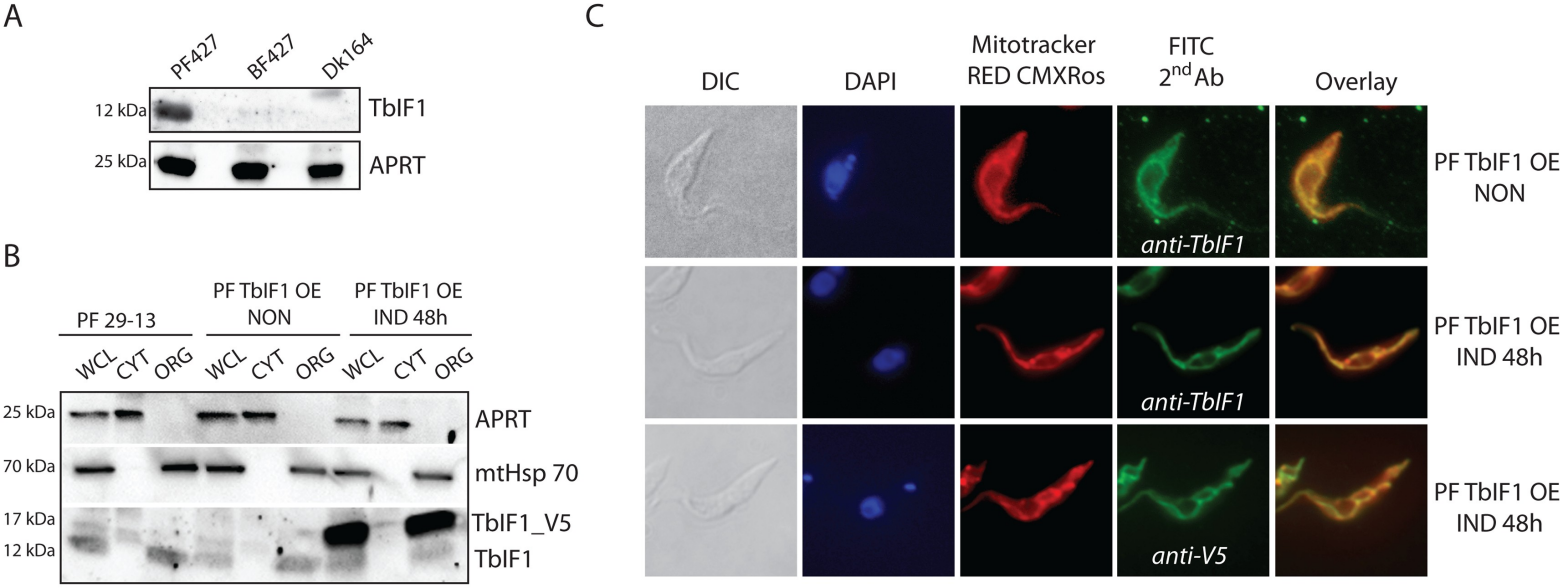
TbIF1 expression is only detected in PF *T*. *brucei*, where it is localized to the mitochondrion. (A) The steady state abundance of TbIF1 was determined in *T*. *brucei* PF427, BF427 and Dk164 whole cell lysates by western blot analysis using a specific polyclonal anti-TbIF1 antiserum. An anti-APRT1 antiserum was used to estimate equal protein loading on SDS-PAGE. The molecular weight of the detected proteins is indicated on the left. (B) TbIF1 subcellular localization was determined in PF 29–13 and PF TbIF1 OE cells either noninduced (NON) or expressing V5-tagged TbIF1 for 48 hours (IND 48h). Whole cell lysates (WCL) and digitonin extracted cytosolic (CYT) and organellar (ORG) fractions were analyzed by immunoblot with the following antibodies: anti-APRT (cytosol), anti-mtHsp70 (organellar fraction), anti-V5 and anti-TbIF1. (C) Immunofluorescence assays with a fluorescein isothiocyanate (FITC)-conjugated secondary antibody that recognizes primary antibodies detecting either all TbIF1 variants (anti-TbIF1) or just the ectopic V5-tagged TbIF1 (anti-V5) further verify that the protein is targeted to the mitochondrion in PF TbIF1 OE cells induced for 48 hours (IND 48h). Noninduced (NON) PF TbIF1 OE cells were included as a control, while the DNA contents and single reticulated mitochondrion were visualized using DAPI (4,6-diamidino-2-phenylindole) and MitoTracker Red CMXRos staining, respectively. The overall cell morphology is depicted in the differential interference contrast (DIC) microscopy images.

To experimentally characterize this putative inhibitory peptide, we first determined its expression in cultured wildtype *T*. *brucei* Lister PF427 and BF427 strains and in the laboratory-induced dyskinetoplastic Dk164 *T*. *brucei* cells. Whole cell lysates harvested from an equivalent number of PF427, BF427 and Dk164 cells were resolved by SDS-PAGE and analyzed by western blot using a specific polyclonal antiserum raised against TbIF1 (Fig 2A). While there was robust TbIF1 expression in PF427 cells, this signal was not detected in the whole cell lysates from BF427 and Dk164 cells. Since we have previously observed that the expression of a purine salvage protein, adenine phosphoribosyl transferase (APRT), does not vary between the trypanosome life stages [46], it was used as a loading control (Fig 2A). These results suggest that one facet of regulating TbIF1 activity includes protein expression levels, since the inhibition of the F_1_-ATPase would be lethal for BF and Dk cells.

To determine the localization of TbIF1, we used PF 29–13 and PF TbIF1 overexpressing (OE) cell line capable of regulating the expression of an ectopic TbIF1 gene product that contained a C-terminal V5-tag. The cytosolic and organellar fractions were isolated from these cells after they were permeabilized with 0.015% digitonin. The subsequent western analyses established the purity of the extracted fractions as the cytosolic APRT and mtHsp70 protein were confined within their respective subcellular fractions. Notably, the endogenous TbIF1 (12 kDa) and its V5-tagged version (17 kDa) were exclusively localized in the organellar fraction (Fig 2B). Furthermore, immunofluorescence assays with either polyclonal TbIF1 antiserum or a monoclonal V5 antibody demonstrated colocalization with the mitochondrion-specific dye, Mitotracker RED CMXRos, which illuminates the typical reticulated structure of a procyclic mitochondrion (Fig 2C).

### Neither TbIF1 silencing nor overexpression produces a negative effect on PF *T*. *brucei* cell growth

**Fig 3:**
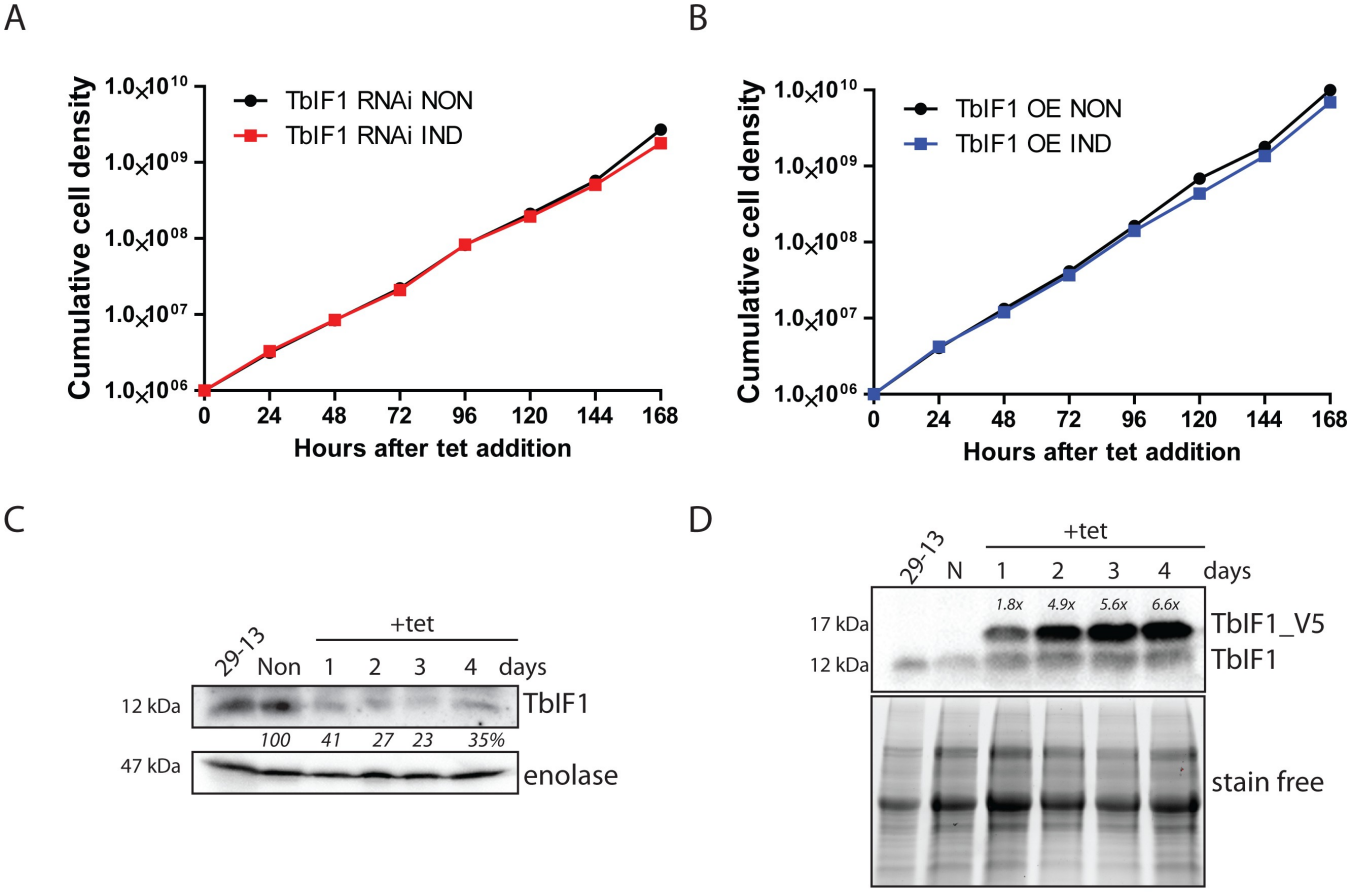
Neither TbIF1 silencing nor overexpression are harmful to PF *T*. *brucei* cells grown *in vitro*. (A) TbIF1 RNAi noninduced (NON) and induced (IND) cells were maintained in the exponential growth phase (between 10^6^ and 10^7^ cells/ml) and the cumulative cell number represents the normalization of cell densities by factoring in the daily dilution factor. The figure is representative of three independent RNAi inductions. B) The growth rate of cells either induced (IND) or noninduced (NON) for TbIF1 OE were determined in the same manner as in A. C) The steady-state abundance of TbIF1 in the parental cell line (29–13), TbIF1 RNAi noninduced (NON) and cells induced (IND) with tet for 1, 2, 3 and 4 days was determined by western blot analysis using a specific TbIF1 antiserum. Cytosolic enolase served as a loading control. The numbers depicted underneath the top panel represent the abundance of immunodetected protein as a percentage of the noninduced samples after normalizing to the loading control. D) Ectopic V5-tagged TbIF1 expression was confirmed by western blot analysis using whole cell lysates from PF 29–13, noninduced (NON) TbIF1 OE and cells induced (IND) for 1, 2, 3 and 4 days. The endogenous TbIF1 and the V5-tagged ectopic protein were visualized using a polyclonal TbIF1 antiserum. Comparable loading was confirmed by Bio-Rad TGX stain-free technology. Levels of V5-tagged TbIF1 overexpression as compared to the endogenous TbIF1 are indicated at the top of the immunoblot.

Due to the expression pattern of TbIF1, we first evaluated its importance in the PF life stage of *T*. *brucei*. In addition to the PF TbIF1 OE cell line, we also created PF cells that silence TbIF1 expression by RNA interference (TbIF1 RNAi). Neither the knockdown of endogenous TbIF1 nor the overexpression of its V5-tagged version resulted in a significant growth phenotype (Fig 3A and 3B). This indicates that TbIF1 is not essential under regular *in vitro* growth conditions and its overexpression doesn’t have a negative effect on PF vitality. The knockdown of TbIF1 was confirmed by western blot analysis of whole cell lysates from TbIF1 RNAi cells that were induced throughout the RNAi time course or never induced (Fig 3C). The polyclonal TbIF1 antiserum revealed a 59% and 73% reduction of the targeted protein on day 1 and 2 of tet addition, respectively (Fig 3C). The partial recovery of the TbIF1 abundance levels on day 4 (35%) might be due to the emergence of RNAi revertants, which is a commonly observed phenomena in *T*. *brucei* cells [47]. Whole cell lysates from PF TbIF1 OE noninduced and induced cells were also analyzed by the same method to confirm the overexpression of the TbIF1-V5 protein (Fig 3D). A polyclonal TbIF1 antiserum detected endogenous TbIF1 in all protein samples, while the ectopic V5-tagged TbIF1 was detected only in tet induced WCL. Bio-Rad TGX stain-free precast gel technology demonstrated comparable loading between samples. Scanning densitometry was then employed to quantify the ratio between ectopic and endogenous TbIF1 in each sample, revealing that protein induction resulted in 1.8 times more V5-tagged TbIF1 on day 1 and up to 6.6 times more on day 4 (Fig 3D).

### TbIF1 prevents the F_o_F_1_-ATPase from establishing a modified Δψm when respiration is chemically inhibited

Since TbIF1 expression levels don’t affect the growth rate of PF *T*. *brucei* grown under normal culture conditions, we sought to characterize the function of TbIF1 when the cells were stressed by chemical hypoxia. While the Δψm in this life stage is normally maintained by the typical cytochrome-containing respiratory complexes III and IV, we wanted to determine if the rotation of the rotary F_o_F_1_ complex can be reversed to maintain the Δψm once the respiratory chain is inhibited. Therefore, we estimated the Δψm in digitonin permeabilized PF *T*. *brucei* cells by utilizing the dye safranin O, a lipophilic cationic dye whose fluorescence becomes quenched when it accumulates within energized mitochondria [48]. The addition of sodium cyanide (NaCN), a potent inhibitor of the respiratory chain, causes the Δψm to dissipate at a rate determined by the speed of a proton leak opposed to the reverse activity of F_o_F_1_-ATP synthase. Under these conditions, we could examine the impact of TbIF1 on this proton-pumping enzyme by observing the changes in these rates when TbIF1 is depleted.

**Fig 4:**
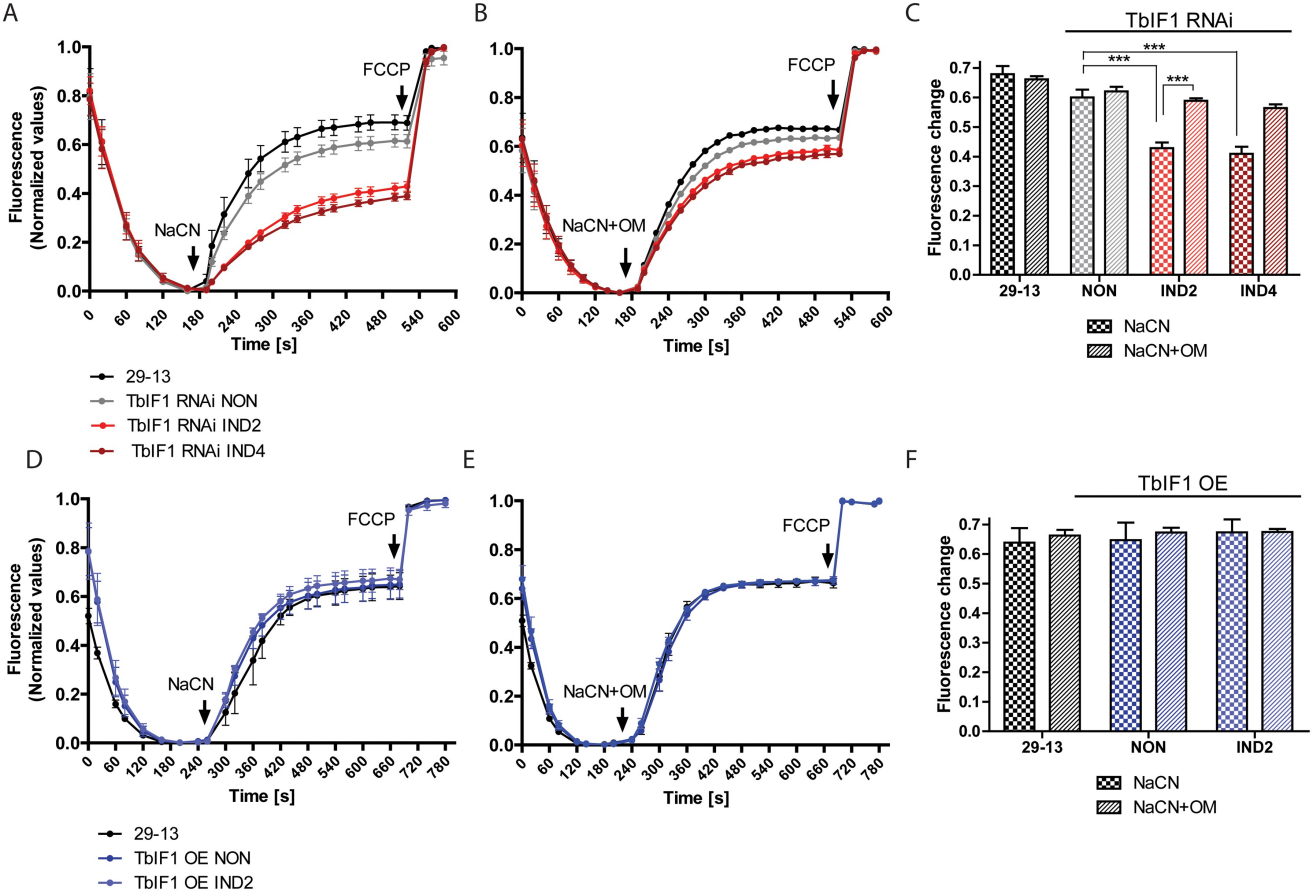
Upon chemical inhibition of respiration, TbIF1 prevents the establishment of a new F_o_F_1_-ATPase mediated Δψm. (A) and (D) The *in situ* dissipation of the Δψm in response to chemical treatment by NaCN was measured using the safranine O dye in the following cell lines: 29–13 (black line), TbIF1 RNAi noninduced (NON, grey line), TbIF1 RNAi induced for 2 and 4 days (IND2 and IND4, red lines), TbIF1 OE noninduced (NON, dark blue line) and TbIF1 OE induced for 2 days (IND2, light blue line). The reaction was initiated with digitonin (50 μM), whereas NaCN (50 μM) and FCCP (20 μM) were added when indicated. (means ± s.d.; n = 3). (B) and (E) The assay described for (A) and (D) was used to observe the dissipation of the Δψm when the same cells were simultaneously treated with 50 μM NaCN and 2.5 μg/ml oligomycin (NaCN+OM). (means ± s.d.; n = 3). (C) and (F) Changes in safranine O fluorescence after the addition of either NaCN or NaCN+OM to the cell lines outlined above. *** *p* < 0.0002, Student’s *t* test.

The parental 29–13 PF trypanosomes served as a positive control to establish the rate of safranine O quenching by energized mitochondria. A baseline for the rapid Δψm depolarization was established when NaCN was injected into the same sample (Fig 4A, black line). Finally, the addition of FCCP, a proton uncoupler, caused the Δψm to completely collapse. A similar pattern depicting the depolarization of the Δψm was observed for TbIF1 RNAi noninduced cells. However, the extent of the Δψm depolarization was significantly smaller in TbIF1 RNAi cells induced for 2 and 4 days. The resulting Δψm was sustained at a new steady state, which is most likely attributable to the F_o_F_1_-ATPase activity (Fig 4A, red lines and Fig 4C). To confirm that the proton-pumping activity of this complex was indeed responsible for this phenotype, 29–13 cells and TbIF1 RNAi noninduced and tet induced cells were permeabilized, allowed to establish a Δψm and then treated with a mixture of NaCN and oligomycin, an inhibitor of F_o_F_1_-ATP synthase. Under these conditions, there were no significant differences observed between the Δψm depolarization rates of these samples (Fig 4B and 4C). These data validate that TbIF1 functions to prevent the reversal of F_o_F_1_-ATP synthase and limit ATP hydrolysis when respiration is disrupted in the parasite.

In a study involving mammalian cells [27], it was presented that the extent of the Δψm depolarization is even greater in cells overexpressing IF1. However, we did not observe this outcome as the rate of the Δψm dissipation remained similar upon NaCN treatment, with or without oligomycin, when we compared either noninduced or induced PF TbIF1 OE cells with the parental line (Fig 4D–4F). This suggests that the abundance of endogenous TbIF1 is sufficient to inhibit the mitochondrial levels of F_o_F_1_-ATPase and thus avert the depletion of ATP under chemically induced hypoxic conditions.

### Overexpression of TbIF1 in PF *T*. *brucei* cells affects the total ATPase activity but not ATP production

**Fig 5:**
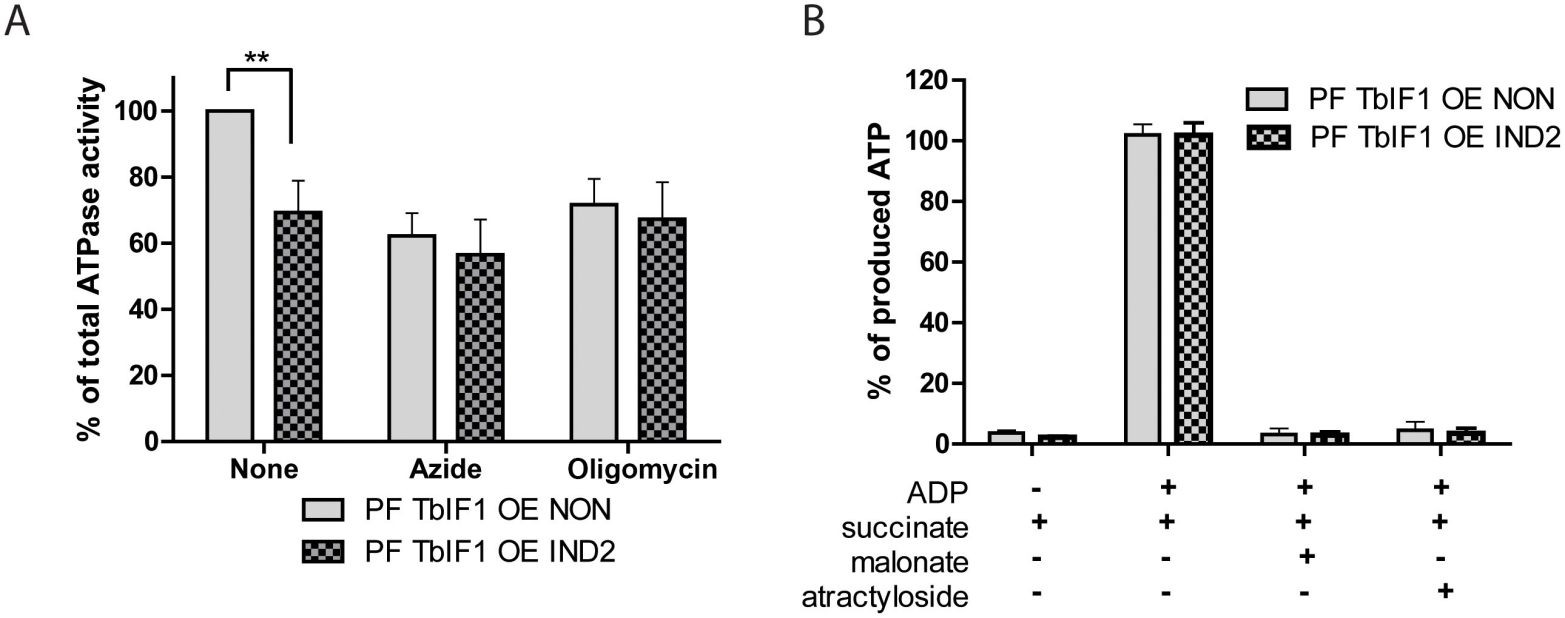
TbIF1 functions as a unidirectional inhibitor of *T*. *brucei* F_o_F_1_-ATP synthase. (A) Total ATPase activity was measured in TbIF1 OE cells that were noninduced (grey) or induced for 2 days with tet (grey cross-hatch). To define the contribution of F_o_F_1_-ATPase to the total ATPase activity measured, samples were also incubated with either azide (AZ, 1 mM) or oligomycin (OM, 2.5 μg/ml). The total amount of free-phosphate detected in the untreated noninduced sample was set at 100%. (means ± s.d.; n = 3; ** *p* < 0.001; Student’s *t* test). (B) The amount of ATP synthesized by oxidative phosphorylation was measured in the digitonin-extracted mitochondria from both noninduced (NON) TbIF1 OE cells and cells induced for 2 days (IND2). The reaction was started by the addition of succinate and ADP. Malonate (mal.) and atractyloside (atract.) were added as specific inhibitors of succinate dehydrogenase and the ATP/ADP carrier, respectively.

Numerous studies demonstrate that yeast and bovine IF1 is a unidirectional inhibitor that impedes the F_o_F_1_-ATPase direction of rotation without hindering the ATP synthesis activity of this complex [25]. Employing the PF TbIF1 OE cell line again, we were able to determine if this unique attribute also applies to *T*. *brucei* cells. The total ATPase activity in mt lysates was indirectly measured by spectrophotometrically detecting the inorganic phosphate that is released during the hydrolysis of ATP by ubiquitous ATPases. Azide (1 mM) and oligomycin (2.5 μg/ml) were used to discern the proportion of the detected ATPase activity that is attributed to F_o_F_1_-ATPase, which typically represents ~ 35–45% of total mt ATPase activity. Importantly, the overexpression of TbIF1 for 2 days resulted in decreased values of total ATPase activity that are comparable to the levels obtained when noninduced TbIF1 OE cells are treated with inhibitors of the rotary enzyme (Fig 5A). Furthermore, since the addition of either azide or oligomycin to samples containing overexpressed TbIF1 doesn’t augment the phenotype, it indicates that TbIF1 specifically inhibits F_o_F_1_-ATPase (Fig 5A).

To assess the potential impact of TbIF1 on the traditional activity of F_o_F_1_-ATP synthase, we examined the ability of the same isolated mitochondria to produce ATP by oxidative phosphorylation. When incubated in a buffer containing ADP, free phosphate and the electron donor succinate, the mitochondria become energized and activate the oxidative phosphorylation pathway to produce ATP, which can be subsequently measured using a bioluminescent substrate. A comparison of the detected ATP levels between noninduced and induced TbIF1 OE cells revealed no major differences in the amount of ATP synthesized (Fig 5B). Additional controls containing malonate and atractyloside, the respective inhibitors of succinate dehydrogenase and the ADP/ATP translocator, were included to verify that the measured ATP truly resulted from oxidative phosphorylation. To define the baseline ATP levels produced from any of the remaining endogenous ADP molecules, a set of samples contained no additional ADP (Fig 5B). Altogether, it seems that under these conditions and elevated expression levels, TbIF1 is only a unidirectional inhibitor that prevents ATP hydrolysis, but does not interfere with ATP synthesis by F_o_F_1_-ATP synthase.

### Overexpression of TbIF1 is lethal to BF and Dk cells

**Fig 6:**
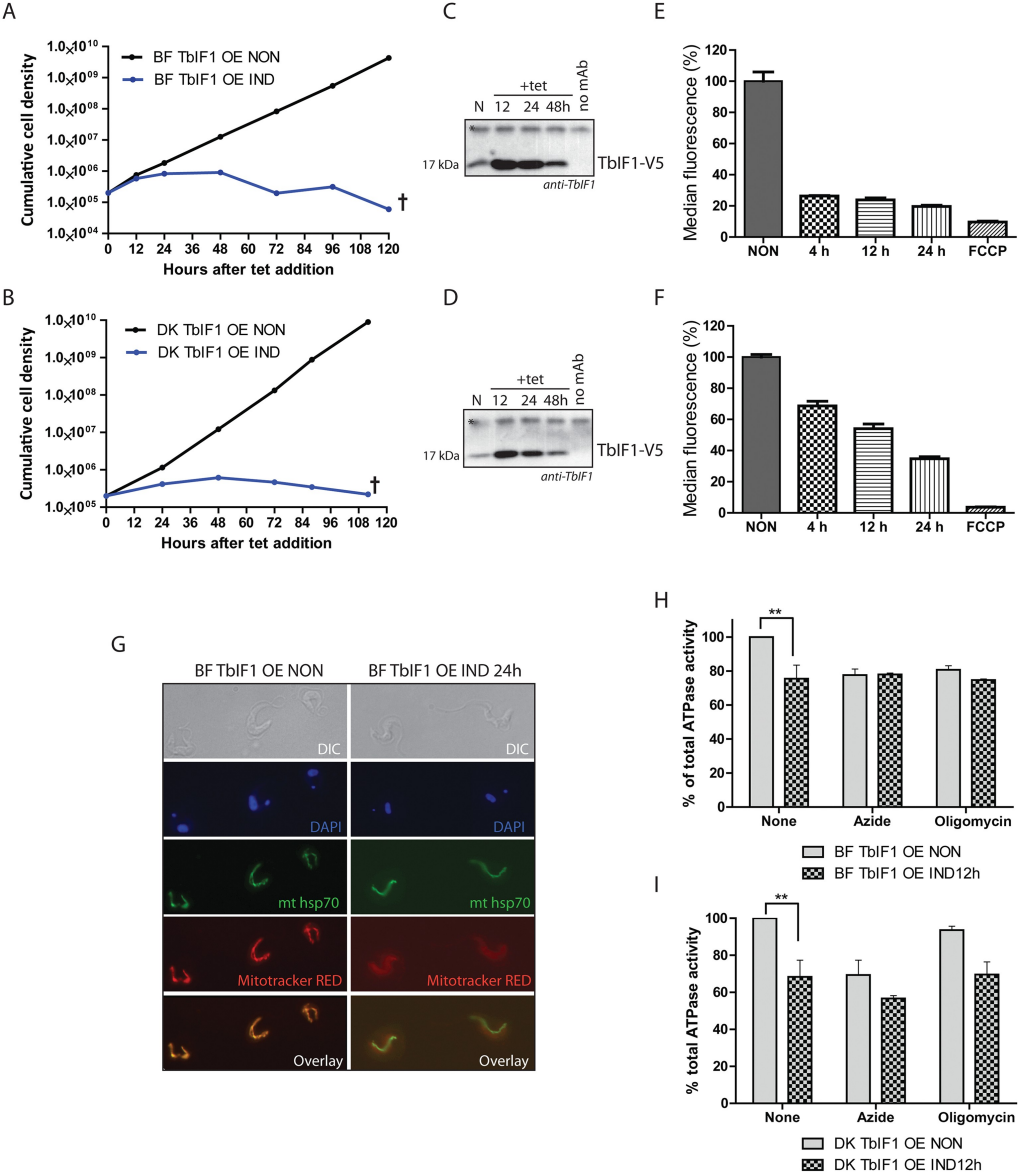
Overexpression of TbIF1 significantly affects the viability of BF and Dk cells due to the dramatic decrease of the Δψ_m_ when F_o_F_1_-ATPase is inhibited. (A, B) Growth curves of noninduced and tet induced BF (A) and Dk (B) TbIF1 OE cells. Cultures were maintained in the exponential growth phase (between 10^5^ and 10^6^ cells/ml) and the figure is representative of three independent tet inductions. (C, D) Western blot analysis of TbIF1_V5 immunoprecipitated with a V5 monoclonal antibody from BF (C) and Dk (D) TbIF1 OE cells that were noninduced (N) or induced for 12, 24 or 36 hours. The purified protein samples were probed with a polyclonal antiserum raised against TbIF1 and the asterisk identifies a nonspecific band that serves as a loading control. The no mAb lane represents nonspecific proteins isolated during an immunoprecipitation step that omitted the V5 antibody. (E, F) Using flow cytometry and the fluorescent dye TMRE, the *in vivo* Δψ_m_ was measured in BF (E) and Dk (F) TbIF1 OE cells that were either not induced or induced for 4, 12 or 24 hours. A noninduced sample was also treated with FCCP to demonstrate that the assay was specifically measuring the Δψm. (means ± s.d.; n = 3). (G) Immunofluorescence of noninduced (NON) or induced (IND 24h) BF TbIF1 OE cells reveals an unchanged overall mt morphology upon TbIF1 induction. Mitochondria were visualized by immunostaining with an anti-mtHsp70 antibody (green) and by staining with MitoTracker Red CMXRos (red), a fluorescentdye that is Δψ_m_ dependent. DNA contents are stained with DAPI (blue) and the morphology of the cells was visualized using DIC imaging. (H, I) Total ATPase activity was quantified in crude mitochondrial preparations isolated from BF (H) and Dk (I) TbIF1 OE cells that were tet induced for 12 hours or not at all. Replicates of these samples were also treated either with oligomycin (OM, 2.5 μg/ml) or azide (AZ, 1 mM) to determine the proportion of this activity generated by F_o_F_1_-ATPase. (means ± s.d.; n = 3; ** *p* < 0.01; Student’s *t* test)

While TbIF1 overexpression does not impair PF growth rate, a quite different outcome was anticipated in BF and Dk cells, since their survival depends on ATP hydrolysis by F_o_F_1_-ATPase to generate the essential Δψm. Indeed, the induced expression of TbIF1 proved to be deleterious to BF and Dk cells, with a strong growth phenotype detected within the first 24 hours of adding tet (Fig 6A and 6B). Interestingly, the V5-tagged TbIF1 was at first not detectable when only whole cell lysates were analyzed by western blot. Thus, an anti-V5 mouse antibody was used to immunoprecipitate the tagged TbIF1, which was then immunodecorated with a TbIF1 rabbit antiserum. This enrichment allowed us to load approximately 40 times more antigen and confirm that very low levels of TbIF1-V5 were expressed upon tet induction (Fig 6C and 6D). Possibly, TbIF1 is so toxic that only cells harbouring a mutation for minimal ectopic expression were selected from the transfection population.

### TbIF1 diminishes the mt Δψm by inhibiting F_1_-ATPase activity in BF and Dk cells

To determine if the overexpressed TbIF1 functioned as expected, the *in vivo* Δψm was measured in these verified cell lines. Flow cytometry analyses measured the changes observed in BF and Dk TbIF1 OE noninduced and induced cells stained with TMRE, whose fluorescence intensity is proportionally dependent on the magnitude of the membrane polarization. After only 4 hours of tet induction, there was already a substantial Δψm decrease (by 73%) in the BF TbIF1 OE cell population that remained low throughout the course of the experiment (Fig 6E). Intriguingly, while the Δψm of the Dk TbIF1 OE cells eventually diminished to similar levels (64% decrease) by 24 hours of TbIF1 expression, the observed decline was more gradual (Fig 6F). This phenotypic pattern might reflect the contrasting contributions of the F_o_F_1_-ATPase to the Δψm in BF and Dk mitochondria. Whereas this molecular machine in BF cells can translocate approximately three protons from the hydrolysis of one ATP molecule, its efficiency is significantly reduced in Dk parasites because it merely supplies the ADP substrate for the electrogenic exchange of ADP^3-^/ATP^4-^ by AAC [34]. Finally, since it is not possible to quantify the absolute Δψm using this assay, it is possible that in Dk cells the Δψm is maintained at a lower level than in BF mitochondria and thus does not require as dramatic of a collapse to produce a detrimental effect.

Notably, we observe a sudden and robust decrease in the Δψ_m_ within the first 24 hours, but there is a lag before the induced BF TbIF1 OE cell culture transitions from an arrested cell division fate to a lethal phenotype. Therefore, the mitochondria of these cells were labelled with Mitotracker Red CMXRos dye, a marker for energized mitochondria, before and after TbIF1 induction. While fluorescence microscopy detected the resulting red dye in noninduced cells, there was no visible staining in cells expressing V5-tagged TbIF1 for 24 hours. However, when the mitochondria of these induced cells are immunostained with a monoclonal anti-mtHsp70 antibody, the overall mt morphology seems to be unaffected (Fig 6G). This suggests that while the Δψ_m_ is required for cell replication, the lethality of TbIF1 overexpression is due to loss of mt protein import [49].

Similar to the results described for PF TbIF1 OE cells, inducing the inhibitory peptide in BF and Dk TbIF1 OE cells decreased the total ATPase activity by 25% and 32% in BF and Dk cells, respectively (Fig 6H and 6I). This correlates nicely with the decreased values obtained for noninduced BF (23%) and Dk (31%) cells treated with azide (Fig 6H and 6I). The potency of this natural inhibitor is further exemplified when compared to the 20% decrease in ATPase activity measured for BF TbIF1 OE noninduced cells treated with oligomycin (Fig 6H). The lack of oligomycin sensitivity observed in Dk TbIF1 OE noninduced cells (Fig 6I) is expected since they are without the mt encoded subunit a, which contains the binding site of this molecular inhibitor [34].

### Recombinant TbIF1 inhibits the F_o_F_1_-ATPase activity in PF *T*. *brucei* mitochondria lysates

**Fig 7:**
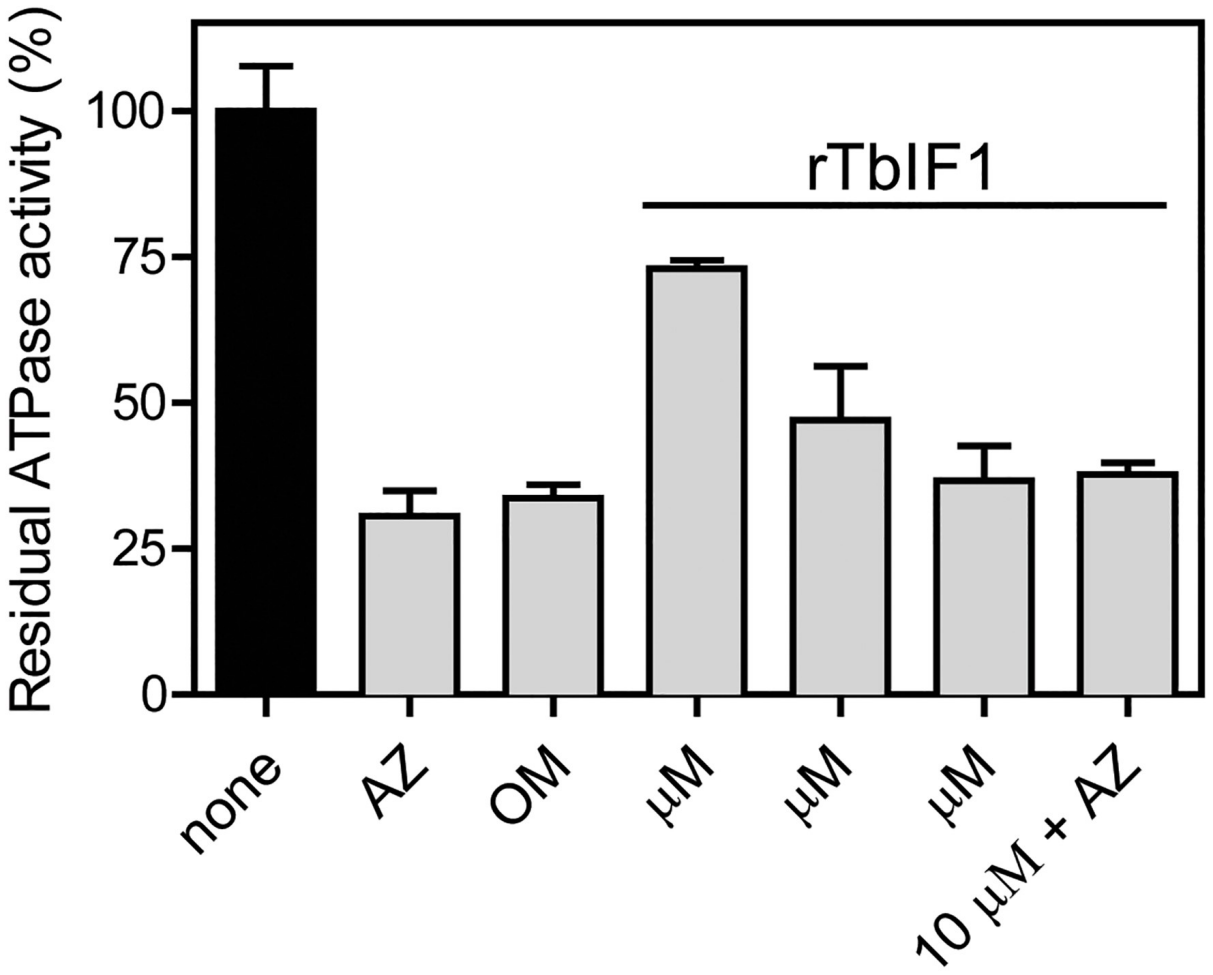
Recombinant TbIF1 inhibits the F_o_F_1_-ATPase activity *in vitro*. Mitochondria isolated from wildtype PF427 cells were lysed with dodecyl maltoside and the ATPase activity was measured by a Pullman assay. These samples were either treated with azide (AZ, 2 mM), oligomycin (OM, 50 μM) or the indicated rTbIF1 concentrations. (means ± s.d.; n > 3).

To directly demonstrate the ability of TbIF1 to inhibit F_o_F_1_-ATPase, rTbIF1 containing a C-terminal hexahistidine tag was expressed in bacteria, purified by nickel affinity chromatography and then incubated with isolated mitochondria (Fig 7). Titrating known amounts of rTbIF1 to a constant volume of a percoll purified mt extract decreased the total ATPase activity in a manner dependent on the concentration of the added inhibitor. The highest input of the recombinant protein (10 μM) caused a 64% drop in activity, which corresponds to the values acquired with azide and oligomycin. Importantly, treating mitochondria with a mixture of both rTbIF1 and azide did not result in an increased inhibition of the total ATPase activity, suggesting that rTbIF1 specifically targets only F_o_F_1_-ATPase.

In summation, these results validate that we have identified the endogenous TbIF1, which is a potent and specific inhibitor of the essential F_o_F_1_-ATPase activity in the BF and dyskinetoplastic trypanosomes.

## Discussion

Under normal physiological conditions, the F-ATP synthase is a nanomotor that synthesizes ATP when the rotation of the machine is driven by protons moving down the electrochemical potential created across the biological membranes in bacteria, chloroplast and mitochondria. An inevitable consequence of the bioenergetic properties of this protein complex occurs when the Δψ collapses and the F-ATP synthase shifts to ATP hydrolysis, which provides the energy necessary to pump protons and create a new gradient. This attempt to maintain the essential Δψ exacts a high energetic toll on the cell and cannot be sustained for long. To prevent this wasteful ATP turnover, several different mechanisms have emerged throughout nature, with unique adaptations described in eubacteria, α-proteobacteria and chloroplasts [50–52]. In mitochondria, IF1 represents yet another distinct regulatory protein that interferes with the intrinsic rotational mechanism of the central stalk. Since its discovery in 1963, the function of this protein to mitigate cell injury upon a loss of respiration has been described in many eukaryotic organisms [19–21,44,53]. The identification and characterization of this protein in the early diverging *T*. *brucei* suggests that IF1 inhibition of F_o_F_1_-ATPase has long been the preferred method to regulate this activity in mitochondria.

Typically, the bioenergetics of healthy cells promote ATP synthesis, thus the silencing of IF1 expression does not impact the fitness of mammalian and yeast cells grown *in vitro* [54,55]. Furthermore, this inhibitor is not required for the normal growth and breeding of mice [56]. To determine if TbIF1 functions in a manner similar to its eukaryotic homologs, we generated a cell line that suppresses the inhibitor by RNAi. In agreement, no growth abnormality was observed in PF *T*. *brucei* cells with significantly decreased TbIF1 expression. Nevertheless, the function of this protein became apparent when the Δψ_m_ was monitored in *Trypanosoma* cultures chemically treated to inhibit respiration. It was only after the depletion of TbIF1 that the F_o_F_1_-ATPase was able to hydrolyze ATP and stabilize a new, albeit lower, electrochemical potential.

Interestingly, relative to the expression levels of the F_1_-ATPase β subunit, the amount of IF1 fluctuates in a variety of mammalian tissues [26]. Based on IF1 protein levels and the glycolytic capacity of the cell, there are two observed responses when cellular respiration is compromised: either the cell will attempt to endure depolarized mitochondria while sustaining ATP pools (high IF1 expression, low glycolytic capacity, e.g. neurons) or it will consume ATP in order to maintain an acceptable Δψm (low IF1 expression, high glycolytic capacity, e.g. astrocytes). Since the overexpression of TbIF1 in PF *T*. *brucei* treated with chemical hypoxia did not further destabilize the Δψm compared to the parental or noninduced cells, it suggests that wildtype PF parasites possess enough TbIF1 to inhibit a majority of the F_o_F_1_-ATPase activity. Therefore, it seems that PF trypanosome cells have chosen the route of high IF1 expression because their energy metabolism is more dependent on proper mt function. The utility of employing this strategy to protect against hypoxic injury by preserving cellular ATP becomes relevant when these protists enter the tsetse midgut, an environment with extremely low oxygen tension [57,58].

Recently, another possible role for the inhibitory protein emerged from the observation that IF1 expression is strongly up-regulated in several types of cancer cells. It is proposed that in these cells, IF1 plays an important role in the metabolic shift from oxidative phosphorylation to aerobic glycolysis, a process known as the Warburg effect, which promotes cellular proliferation and survival [28]. Similar metabolic adaptations are also observed during the *T*. *brucei* life cycle as procyclic cells transition into a metacyclic stage before they fully convert to the long slender bloodstream form. It is possible that the infective metacyclic trypanosomes biding their time in the salivary glands before they are transmitted to the glucose-rich bloodstream of the mammalian host have already re-programed their metabolism and are primed for enhanced aerobic glycolysis. We are now exploring if TbIF1 contributes to this remarkable cellular transformation using an *in vitro* differentiation system [59].

Numerous genome-wide studies have previously shown that TbIF1 expression is strongly regulated between PF and BF parasites. Since TbIF1 mRNA is strongly down-regulated in bloodstream cells, its half-life was impossible to measure and was therefore assigned to the class of “procyclic form specific” mRNAs. This subset of molecules consists of only 29 transcripts, which notably includes GPEET2 and EP2 procyclins [60]. Furthermore, it has been reported that TbIF1 transcript levels are already upregulated after just 1 hour of inducing BF cells to transform into PF parasites with citrate/cis-aconitate at 27°C. In fact, TbIF1 expression levels continue to rise throughout this differentiation process [61]. In agreement with the transcriptomic data [62–64], proteomic studies based on SILAC labeling of PF and BF cells confirmed TbIF1 is strongly up-regulated in the insect stage of the parasite [65,66]. The striking difference in TbIF1 expression between the PF and BF life stages illustrates the deleterious effects of TbIF1 when the Δψ_m_ is maintained by F_o_F_1_-ATPase. It also provides a nice model to study gene expression in an organism that predominantly regulates these processes at the post-transcriptional level.

While the activity of the mitochondrion is reduced during the infectious stage, several biological processes unique to *T*. *brucei* have previously been identified as potential drug targets within this organelle (alternative electron transport chain, kDNA and topoisomerases, tRNA import and fatty acid synthesis) [67]. The F_o_F_1_-ATPase in particular is an appealing target as it is required for maintaining the essential Δψ_m_ in BF trypanosomes, while the mammalian host cells almost exclusively rely on this nanomotor to synthesize ATP. In fact, it has been demonstrated that trypanocides belonging to two different classes of drugs, the aromatic diamidines (i.e., DB75) and bisphosphonate salts (i.e., AHI-9), can inhibit the F_o_F_1_-ATPase activity [68,69]. Unfortunately, it appears that these compounds are likely promiscuous inhibitors that not only interact with multiple cellular targets, but also indiscriminately inhibit both the ATP hydrolytic and synthetic activities of the enzyme. Furthermore, since the F_o_F_1_-ATPase binding sites and the exact mechanism of inhibition for these molecules would be challenging to ascertain, it would require significant effort to improve their efficacy. Since TbIF1 is a specific and unidirectional inhibitor of the *T*. *brucei* F_o_F_1_-ATPase, we can now exploit the intrinsic binding properties of this endogenous inhibitor to facilitate future structure-based drug design. Specifically, the development of peptidomimetics [70–73] that simulate the most important binding interactions of TbIF1 to *T*. *brucei* F_1_-ATPase could potentially result in effective therapeutics that incapacitate these medically and economically important parasites.
